# LRRK2 mediated Rab8a phosphorylation promotes lipid storage

**DOI:** 10.1186/s12944-018-0684-x

**Published:** 2018-02-27

**Authors:** Miao Yu, Muhammad Arshad, Wenmin Wang, Dongyu Zhao, Li Xu, Linkang Zhou

**Affiliations:** 1 MOE Key Laboratory of Bioinformatics and Tsinghua-Peking Center for Life Sciences, School of Life Sciences, Tsinghua University, Beijing, 100084 China; 2 Department of Bioinformatics and Biotechnology, International Islamic University, Islamabad, 44000 Pakistan

**Keywords:** LRRK2, Rab8a, Lipid droplets, Lipid storage, Parkinson’s disease

## Abstract

**Background:**

Several mutations in *leucine rich repeat kinase 2* (*LRRK2*) gene have been associated with pathogenesis of Parkinson’s disease (PD), a neurodegenerative disorder marked by resting tremors, and rigidity, leading to Postural instability. It has been revealed that mutations that lead to an increase of kinase activity of LRRK2 protein are significantly associated with PD pathogenesis. Recent studies have shown that some Rab GTPases, especially Rab8, serve as substrates of LRRK2 and undergo phosphorylation in its switch II domain upon interaction. Current study was performed in order to find out the effects of the phosphorylation of Rab8 and its mutants on lipid metabolism and lipid droplets growth.

**Methods:**

The phosphorylation status of Rab8a was checked by phos-tag gel. Point mutant construct were generated to investigate the function of Rab8a. 3T3L1 cells were transfected with indicated plasmids and the lipid droplets were stained with Bodipy. Fluorescent microscopy experiments were performed to examine the sizes of lipid droplets. The interactions between Rab8a and Optineurin were determined by immunoprecipitation and western blot.

**Results:**

Our assays demonstrated that Rab8a was phosphorylated by mutated LRRK2 that exhibits high kinase activity. Phosphorylation of Rab8a on amino acid residue T72 promoted the formation of large lipid droplets. T72D mutant of Rab8a had higher activity to promote the formation of large lipid droplets compared with wild type Rab8a, with increase in average diameter of lipid droplets from 2.10 μm to 2.46 μm. Moreover, phosphorylation of Rab8a weakened the interaction with its effector Optineurin.

**Conclusions:**

Y1699C mutated LRRK2 was able to phosphorylate Rab8a and phosphorylation of Rab8a on site 72 plays important role in the fusion and enlargement of lipid droplets. Taken together, our study suggests an indirect relationship between enhanced lipid storage capacity and PD pathogenesis.

## Background

Rabs are a group of about 70 eukaryotic proteins located on different membranes that play important roles in all stages of vesicle trafficking including budding, motility and fusion [1, 2]. The most important feature of Rab proteins is that they can switch from inactive GDP-bound form to active GTP-bound form and recruit unique effectors which play their roles in vesicle trafficking [3]. The binding of GTP or GDP changes the conformation in Rab Switch I and Switch II zones which are crucial for maintaining the specific conformation of Rab proteins. The switching of Rab GTPases is mediated by four classes of proteins and the inhibition of Rab pathway results in diseases like immunodeficiency or neurological disorders [4]. Many Rabs can undergo phosphorylation on serine or threonine residues [5]. Rab5a has been reported to be phosphorylated by PKC (Protein kinase C) on Thr (threonine)-7 site. Rab5a phosphorylation is functionally necessary for Rac1 activation, actin rearrangement and T-cell motility [6]. Rab24 can undergo tyrosine phosphorylation and this modification may influence Rab24 targeting and interactions with effector protein complexes [7].

Rab8 has shown to play important roles in membrane trafficking. It plays irreplaceable roles in multiple biological processes including cellular morphology, cell polarity, cell movement, neural differentiation and ciliogenesis [8]. Knocking down of Rab8 or its effector Optineurin results in the inhibition of transportation of transferrin protein from membrane to intracellular part while transferrin protein receptor fails to return to ERC (endocytic recycling compartment) [9, 10]. Furthermore, Rab8 is recruited by Rab6 onto the vesicles responsible for exocytosis where it controls exocytosis activity [11, 12]. Rab8 could also regulate the fusion between vesicles and cell membrane [13].

LRRK2 (leucine rich repeat kinase 2) has been a hot spot in the field of neurology as its mutations have been associated with Parkinson’s disease (PD) [14, 15]. PD is a common neurodegenerative disease affecting approximately 1% of the elderly population and is marked by resting tremors, rigidity, and akinesia [16, 17]. The progression of the disease results in postural instability. While the causative mechanisms of PD remain elusive, the findings that more than twenty mutations on *LRRK2* gene are related to PD have brought LRRK2 into main focus of research [18]. LRRK2 is a large protein consisting of central kinase and GTPase domains which are surrounded by several protein-protein interaction regions. PD pathogenic *LRRK2* mutations (G2019S, I2020T, R1441C/G/H, Y1699C) are primary causes for PD pathogenesis. Till now, it remains unclear how *LRRK2* mutations occurring in different functional domains predispose to PD. The most powerful PD-associated *LRRK2* mutation is G2019S, which increases LRRK2 kinase activity two to three fold [19, 20]. Since LRRK2 is a kinase, its substrates may have important role in pathogenesis.

Recently, a subset of Rab GTPases has been identified as key LRRK2 substrates. Through a combination of phosphoproteomics and molecular genetics approaches, it has been proved that several Rab GTPases especially Rab8 can be phosphorylated by LRRK2 on an evolutionary conserved residue in switch II domain [21]. Since switch II domain plays crucial role in maintaining Rab protein conformation, the phosphorylation may alter the behavior of Rab proteins. Considering this, we have tried to find more evidences about the consequences of the phosphorylation of Rab8 and have found out an important relationship between lipid storage and PD pathogenesis.

## Methods

### Cell culture

Two hundred ninety-three T cells and 3 T3-L1 pre-adipocytes were cultured in DMEM (Invitrogen, USA) containing 10% FBS (Invitrogen, USA), 2 mM L-glutamine, 100 U/mL penicillin and 100 μg/ml streptomycin at 37 °C in humidified incubators containing 5% CO_2_. Methods for 3T3L1 preadipocytes differentiation and electrotransfection were similar as previously described [22].

### Cell transfection

DNA plasmids were transfected into 293 T cells and 3 T3-L1 pre-adipocytes using Lipofectamine 2000 following manufacturer’s instructions (Invitrogen, USA).

### Plasmids construction

Human LRRK2 and its mutant (LRRK2 Y1699C) plasmids were purchased from Addgene (code #17609 and #25364). Full-length cDNAs encoding mouse *Rab8a* and *Optinurin* were amplified from cDNA of 3 T3-L1 adipocytes. These cDNAs were subcloned into pCMV5-HA or pCMV5-FLAG using specific restriction sites. Point mutations of *Rab8a* were cloned by a PCR-based site-directed mutagenesis method (Stratagene, USA). The accuracy of each plasmid DNAs was tested by sequence analysis.

### Western blot

Methods for immunoprecipitation and western blot sample preparation were same as previously described [22]. Anti-FLAG M2 agarose beads (cat: A2220) were purchased from Sigma. The antibody against Fsp27 was used as described previously [22]. Antibodies against Actin (A5441, Sigma), FLAG (mouse source, F1804, sigma), MYC (sc-40, Santa Cruz), LRRK2 (5559, Cell signaling), Rab8a (6975, Cell signaling) and HA (sc-7392, Santa Cruz) were used for western blot analysis. The blots were detected using HRP-conjugated secondary antibodies (GE Healthcare, UK) and the ECL-Plus system.

### Phos-tag gel

Phos-tag acrylamide and MnCl_2_ were added to a standard gel solution to the final concentration of 50 mM and 100 mM, respectively. Then gels were polymerized by ammonium per-sulfate and TEMED. Cell lysates used for Phos-tag SDS-PAGE were supplemented with MnCl_2_ at 10 mM to neutralize the effect of EDTA in the lysates. After running SDS-PAGE, gels were washed 3 times with transfer buffer containing 10 mM EDTA followed by a wash with transfer buffer (10 min each). Blotting to nitrocellulose membranes was carried out by following a standard protocol. Phos-tag acrylamide was kept at 4 °C in black tubes blocking out light because Phos-tag acrylamide is light-sensitive.

### Imaging

Images for analyzing lipid droplets sizes were acquired under an LSM710 confocal microscope (Carl Zeiss) with 63× oil immersion objective. Images were exported out in 16-bit TIFF format. Further processing of single images (e.g., amplifying a certain region) was performed in Photoshop (CS2; Adobe).

### Lipid droplet size analysis

Quantitative analysis of LD size in 3 T3-L1 preadipocytes was performed as described by Sun et al. [23]. For quantitative analysis of cells containing large LDs, DNA plasmids were transfected into 3 T3-L1 preadipocytes using Lipofectamine 2000. Cells were incubated with 200 mM OA complexed with albumin for 15 h before they were fixed. The largest LDs’ sizes were measured in each cell. Approximately 150 cells from three independent experiments were analyzed. Images were obtained under an inverted microscope Axiovert.

### Statistics

All statistical analyses were performed in GraphPad Prism Version 5 (GraphPad Software). Significance was established using a two-tailed Student’s *t*-test. Differences were considered significant at *P* < 0.05. *** indicated *P* < 0.001.

## Results

### Rab8a is phosphorylated by LRRK2

**Fig. 1:**
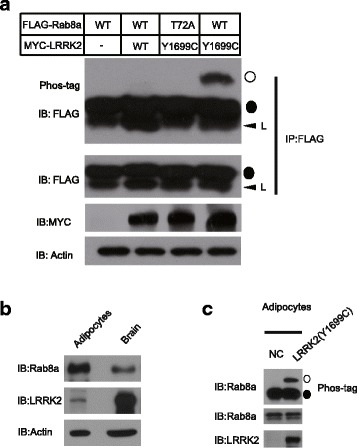
Phos-tag analysis of LRRK2 Y1699C mediated Rab8a phosphorylation. **a** 293 T cells were transfected with MYC-LRRK2 (Wild type or Y1699C), and FLAG-Rab8a (WT or T72A mutant). Anti-FLAG M2 beads were used for immunoprecipitation. The immunoprecipitated products were detected by using antibody against FLAG. Phosphorylation of overexpressed Rab8a was analysed by a Phos-tag assay (top panel). Equal levels of expression of FLAG-Rab8a and MYC-LRRK2 were confirmed by immunoblotting on normal gels using an anti-FLAG (second panel from the top) and anti-MYC (third panel from the top) antibodies respectively. Actin was used as a loading control (bottom panel). L represents light chain. ○ represents phosphorylated Rab8a. ● represents non-phosphorylated Rab8a. Similar results were obtained in at least two separate experiments. **b** The expression level of Rab8a and LRRK2 in adipocytes. **c** The phosphorylation of endogenous Rab8a in adipocytes. Differentiated adipocytes were transfected with LRRK2 (Y1699C)

In order to confirm whether Rab8a could be phosphorylated by LRRK2, we overexpressed FLAG-tagged Rab8a (Wild-type form and T72A form. “T” represent threonine and “A” represent alanine) and MYC-tagged LRRK2 (Wild-type form and Y1699C form) in 293 T cells. The phosphorylation form of Rab8a was detected using phos-tag gel. As shown in Fig. 1a, Y1699C mutation of LRRK2, but not wild-type LRRK2, could phosphorylate Rab8a as indicated by the shift in the band. The T72A mutant of Rab8a could not be phosphorylated by LRRK2 (Y1699C), indicating that the phosphorylation of Rab8a by LRRK2 Y1699C exclusively occurs on T72 site. Next, we wondered whether endogenous Rab8a could be phosphorylated by LRRK2. We first checked the expression level of Rab8a and LRRK2 in differentiated adipocytes. The expression of Rab8a was found to be higher compared with that in brain. The expression of LRRK2 was detected in adipocytes, although the expression level was low compared with that in brain (Fig. 1b). We transfected LRRK2 (Y1699C) in differentiated adipocytes. Then we performed immunoprecipitation of Rab8a using Rab8a antibody, and later ran the phos-tag gel to check the phosphorylation status of Rab8a. Overexpression of LRRK2 in adipocytes could dramatically induce the phosphorylation of endogenous Rab8a (Fig. 1c).

### Phosphorylated Rab8a on site 72 promotes lipid droplets fusion and enlargement

**Fig. 2:**
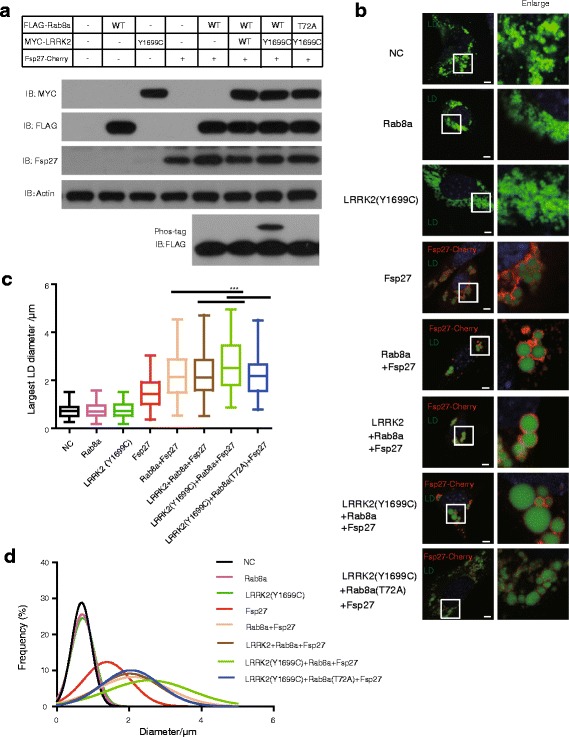
Rab8a phosphorylation promotes lipid droplets enlargement. 3 T3-L1 pre-adipocytes were transfected with MYC-LRRK2 (Wild type or Y1699C), FLAG-Rab8a (WT or T72A mutant) and Fsp27-Cherry. Oleic acid was added to promote the formation of LDs for 15 h. LDs were labeled with Bodipy 493/503 (Green). **a** Expression levels of LRRK2, Rab8a and Fsp27 were detected by western blot. **b** Co-expression of LRRK2 Y1699C with FLAG-Rab8a increases the sizes of LDs when over-expressing Fsp27. Scale bars represent 20 μm. **c**-**d** Statistical analysis of (**b**). Significance was established using a two-tailed Student’s *t*-test. Differences were considered significant at *P* < 0.05. ***P < 0.001

Our previous data has shown that Rab8a plays important role in Fsp27 mediated lipid droplet fusion and enlargement [22]. Here, we wanted to investigate the role of phosphorylated Rab8a on lipid droplet enlargement. We overexpressed Rab8a (wild-type, T72A), LRRK2 (wild-type, Y1699C) together with Fsp27-Cherry and relative controls in 3 T3-L1 pre-adipocytes and the lipid droplets in the cells were stained with Bodipy 493/503. Overexpression of Rab8a, LRRK2 (Y1699C) could not enhance the size of lipid droplets compared with the controls. However, overexpression of Fsp27 could dramatically induce lipid droplets’ size compared with control cells. Rab8a could enhance Fsp27’s ability in promoting lipid droplets enlargement. Co-overexpression of LRRK2 (Y1699C) and Rab8a resulted in the formation of enlarged lipid droplets compared with Rab8a overexpression alone (Fig. 2a-d), with increase in average diameter of lipid droplets from 2.26 μm to 2.69 μm. However, co-expression of wild-type LRRK2 with Rab8a showed no significant impact on the size of lipid droplets when compared with Rab8a overexpression alone. The size of lipid droplets in cells overexpressing Rab8a (T72A) together with LRRK2 (Y1699C) was similar with the cells overexpressing Rab8a alone. These data indicate that the phosphorylation of Rab8a on site 72 plays important role in the fusion and enlargement of lipid droplets.

### T72D/E form of Rab8a promotes the formation of large lipid droplets

**Fig. 3:**
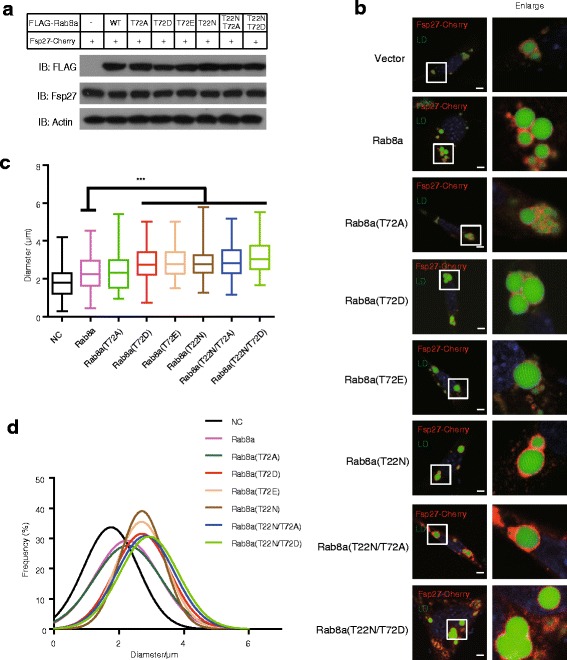
Overexpression of mimic phosphorylated Rab8a (Rab8a T72D and Rab8a T72E) increases the size of LDs. 3T3L1 pre-adipocytes were transfected with FLAG-Rab8a (WT, T72A, T72D, T72E, T22 N, T22 N/T72A, T22 N/T72D) and Fsp27-Cherry. Oleic acid was added to promote the formation of LDs for 15 h. LDs were labeled with Bodipy 493/503 (Green). **a** Western blots showing protein levels of FLAG-tagged Rab8a and its mutations. **b** & **c** Overexpression of Rab8a T72D and T72E increases the sizes of LDs under the circumstances of over-expressing Fsp27 while overexpression of wild-type Rab8a or phosphorylation defective Rab8a (T72A) shows no such effects. Scale bars represent 20 μm. **d** Statistical analysis of (**b**). Significance was established using a two-tailed Student’s *t*-test. Differences were considered significant at *P* < 0.05. ***P < 0.001

Since point mutation from threonine (T) to aspartic acid (D) or glutamic acid (E) can mimic phosphorylation, different mutations on the T72 site of Rab8a were generated (T72A, T72D and T72E). T72A represents the non-phosphorylated form of Rab8a while T72D/E mimics the phosphorylated form of Rab8a. We wanted to investigate the role of above mentioned Rab8a mutants in lipid droplets enlargement. Overexpression of WT Rab8a could enhance Fsp27’s ability to promote the formation of large lipid droplets. Our results showed that overexpression of Rab8a (T72D) and Rab8a (T72E) increased the size of LDs from 2.27 μm to 2.87 μm when compared with overexpression of Rab8a or Rab8a (T72A) (Fig. 3). According to our previous findings [22], the GDP-bound form of Rab8a (T22 N) could significantly enhance the formation of large lipid droplets as compared to wild-type Rab8a. Interestingly, the effects of T72D and T72E resemble the effect of Rab8a (T22 N), indicating that the phosphorylation of Rab8a on site 72 may affect the GTP/GDP binding status of Rab8a. We then introduced T22 N/T72A and T22 N/T72D, two double mutants of Rab8a. The T22 N/T72A mutant demonstrated effect similar to that of T22 N on the size of lipid droplets. The T22 N/T72D mutant showed slightly increased effect in promoting lipid droplets enlargement when compared with T22 N, with an increase in size of lipid droplets from 2.84 μm to 3.17 μm.

**Fig. 4:**
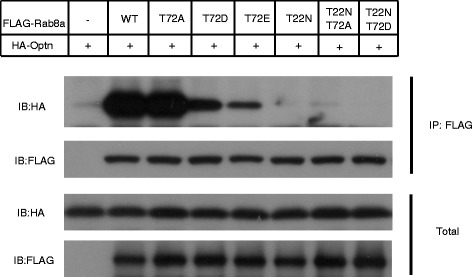
Phosphorylation of Rab8a weakens its interaction with its effector Optineurin. Two hundred ninety-three T cells were transfected with FLAG-Rab8a (WT, T72A, T72D, T72E, T22 N, T22 N/T72A, T22 N/T72D) and HA-Optineurin. Anti-FLAG M2 beads were used for immunoprecipitation. The immunoprecipitated products were detected by using antibodies against FLAG or HA. IP, immunoprecipitation; IB, immunoblot; Western blots showing protein level of HA-Optineurin (top panel) and FLAG-Rab8a (second panel from the top) in the IP products. Western blots showing protein level of HA-Optineurin (third panel from the top) and FLAG-Rab8a (bottom panel) in the total cell lysis

Next, we investigated whether the phosphorylation of Rab8a on 72 site affects its GTP/GDP binding status. Optineurin has been reported to be the effector of Rab8a which binds to the GTP form but not to the GDP form of Rab8a [9]. We co-expressed FLAG-Rab8a and its different mutations (containing wild-type, T72A, T72D, T72E, T22 N, T22 N/T72A, T22 N/T72D) together with HA-Optineurin, then immunoprecipitated down FLAG-tagged Rab8a. As shown in Fig. 4, Rab8a-T22 N showed nearly no interaction with Optineurin compared with wild-type Rab8a. Interestingly, Rab8a-T72D and Rab8a-T72E also showed weak interactions with Optineurin which resembles the Rab8a-T22 N interaction. T22 N/T72A and T22 N/T72D mutants also lost the binding with Optineurin. These results show that phosphorylation of Rab8a weakens its interaction with its effector Optineurin.

## Discussion

In this study, we have shown that Rab8a could be phosphorylated by Y1699C mutated LRRK2. Previous studies showed that there had been an increase in enzyme activity of LRRK2 Y1699C compared with WT LRRK2 [20, 24, 25]. The Rab8a 72 Tyrosine was the only site phosphorylated by mutated LRRK2. We also found out that T72 phosphorylation significantly enhanced the lipid storage activity of Rab8a. Our previous work has already shown that GDP form of Rab8a (T22 N) has higher activity in promoting lipid storage [22]. T72D/E mutation stabilizes the GDP form of Rab8a.

Earlier studies have shown that LRRK2 mutations (Y1699C, G2019S) which increase the activity of LRRK2 are the most common causes of the inherited form of PD pathogenesis [26], however, it is still unclear why over-activation of LRRK2 is harmful and what role it plays inside cells. Moreover, LRRK2 knockout models studies have manifested Rabs as primary substrates of LRRK2 as *LRRK2* knockout rats and mice have shown deformed lungs and kidneys due to possible defects in the autophagosome pathway regulated by activated Rab proteins [27]. Recent studies have shown that both Rab8a and Rab10 are the substrates of LRRK2, indicating that LRRK2-Rabs circuit plays important role in the pathogenesis of PD.

Overexpression of Rab8a, Rab1 and Rab3a proteins alleviate PD through reduced α-synuclein-induced cytotoxicity [28, 29]. As α-synuclein is a lipid droplet bound protein, its dysfunction or overexpression can cause PD as well as lipid droplet formation [30–32]. Some recent studies have identified the presence of lipid droplets in neurons and in glia under certain disease conditions which suggest some association of disrupted lipid-droplet function with neurodegeneration [33, 34]. The size of LDs reflects lipid storage capacity and has been linked to the development of obesity, diabetes, hepatic steatosis and atherosclerosis [35]. The LRRK2-Rab8a mediated LDs enlargement may play important role in PD pathogenesis. Other kinases found mutated in PD pathogenesis, such as PINK1, could also regulate the phosphorylation of Rab8a at site Serine 111 [36]. Some studies have begun focusing on the role of lipid metabolism in PD. A recent study has demonstrated that many sphingolipid, glycerophospholipid and cholesterol species were found altered in the visual cortex of PD patients [37, 38]. Here, we have proposed that Rab8a phosphorylation by LRRK2 alters the ability of lipid storage in PD. Normalization of lipid metabolism may present a novel route for treatment of some types of PD patients.

Another study has established that change in Rab homeostasis may contribute towards development of PD. As LRRK2 is the main regulator of Rab proteins, overactive LRRK2 results in increased Rab phosphorylation, altering the balance between cytosolic and membrane bound Rab, which in turns disturbs intracellular trafficking. In PD-associated LRRK2 mutations, the membrane-cytosol balance of Rabs is shifted towards the membrane causing accumulation of inactive Rabs in the membranes [21]. Moreover, pathogenic LRRK2 mutations outside the kinase domain can also increase Rab phosphorylation which, according to our study, would result in the formation of enlarged lipid droplets, a manifestation of PD pathogenesis.

## Conclusion

Our findings have proved that Y1699C mutated LRRK2, but not wild-type LRRK2, could phosphorylate Rab8a and that phosphorylation of Rab8a on site 72 plays important role in the fusion and enlargement of lipid droplet. Furthermore, our results show that phosphorylation of Rab8a weakens its interaction with its effector Optineurin. It will be interesting to investigate the direct role of lipid droplets accumulation with pathogenesis of PD in order to explore therapeutic interventions against this disease.

## Abbreviations

ERC: Endocytic recycling compartment
LDs: Lipid droplets
LRRK2: Leucine rich repeat kinase 2
PD: Parkinson’s disease
PKC: Protein kinase C
